# *Culex quinquefasciatus* Mosquitoes Exposed to a Juvenile Hormone Analog may Take a Bloodmeal While Gravid

**DOI:** 10.1002/arch.70066

**Published:** 2025-05-24

**Authors:** Grayson A. Tung, Dina M. Fonseca

**Affiliations:** ^1^ Center for Vector Biology Rutgers University New Brunswick New Jersey USA

**Keywords:** behavioral physiology, blood‐feeding, endocrinology, juvenile hormone, nutrition

## Abstract

Blood avidity in female mosquitoes has been shown to be regulated by cycles of hormone production that determine both egg development and distinct behaviors. Specifically, juvenile hormone (JH) drives early egg development until a bloodmeal is acquired, and JH titers are positively correlated with active host seeking and blood feeding behaviors. After a bloodmeal, JH levels fall, and female mosquitoes become refractory to host seeking and biting. While JH analogs (JHAs) are commonly used as larvicides for mosquito control, the effects of these compounds on adult mosquitoes are not well understood. If JH levels are directly implicated in blood acquisition, adult exposure to JHAs might cause nonbiting female mosquitoes to take a blood meal. To test this hypothesis, in laboratory experiments we exposed gravid *Culex quinquefasciatus* mosquitoes to s‐hydroprene, a JHA, both through direct topical application and a simulated environmental exposure. We found a significant increase in the likelihood of gravid *Cx. quinquefasciatus* taking a bloodmeal after exposure to JHAs at levels we hypothesize they may encounter in the field. We also measured the fertility of females that had taken a second bloodmeal while gravid and found a significant negative effect on both the number and hatch rate of eggs. Our results support the expectation that JH levels regulate female blood feeding behaviors. They also suggest that application of JHAs for larval control can unintentionally lead to additional blood feeding events per gonotrophic cycle, with potential increases in the transmission of disease agents.

## Summary

- Mosquitoes exposed to juvenile hormone analog (JHA) blood‐feed while gravid.
- Taking a second bloodmeal while gravid reduces egg number and hatch rate.
- Gravid females are a good model to examine juvenile hormone's effects on behavior.

## Graphical abstract

We applied s‐hydroprene, a juvenile hormone analog (JHA), to gravid female mosquitoes that have naturally ceased JH production. We found that gravid females exposed to JHAs blood fed in a dose dependent manner and this behavior could be induced through simulated environmental exposure. Additionally, we found that taking a bloodmeal while gravid has negative consequences for total reproductive output.

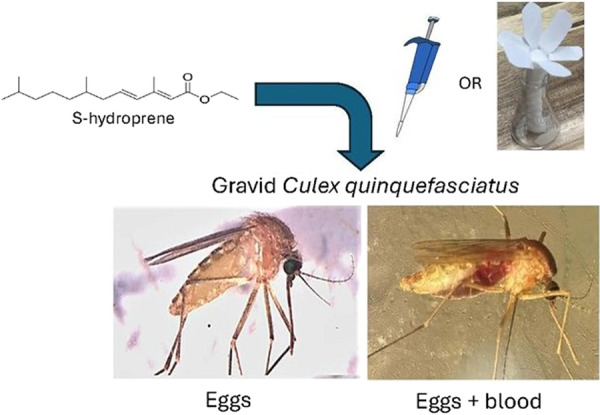

## 1 Introduction

Hematophagy in mosquitoes is a complex behavior modulated by multiple external factors in tandem with internal physiological processes which facilitate the acquisition of blood (Tung and Fonseca 2024). Most female mosquitoes are anautogenous and require blood for the development of viable eggs. Before active host seeking and blood feeding, eggs undergo a period of pre‐vitellogenic egg development, during which oocytes develop competence to uptake nutrients and yolk proteins derived from a future bloodmeal (Zhu and Noriega 2016). In anautogenous females, once oocytes have completed pre‐vitellogenic egg development, eggs cease development until a blood meal is acquired (Raikhel et al. 2002). During this state of arrested egg development female mosquitoes actively seek and bite hosts. After acquiring a bloodmeal, females enter a period of unresponsiveness (refractoriness) to hosts while eggs complete development. Host seeking and biting resume once these eggs have been deposited and a new batch of eggs can be produced. This pattern of egg development and associated behaviors are largely conserved across mosquito taxa with the notable exception of autogenous species that do not require blood (O'Meara 1979; O'Meara and Evans 1977). In addition, under specific environmental stressors such as lack of suitable oviposition sites and dehydration females of some species have been shown to take additional blood meals (Govoetchan et al. 2013; O'Meara 1979; Hagan et al. 2018; Johnson and Fonseca 2014).

Cycles of biting and egg production are regulated by juvenile hormone (JH) production; titers of JH correlate with the discreet periods of egg production, host seeking, and unresponsiveness to hosts (Zhu and Noriega 2016). JH is produced at relatively constant levels until a blood meal is taken at which point JH production ceases. These two patterns of production align with active host seeking and unresponsiveness to hosts, respectively. Additionally, removal of the *corpora allata*, the neuroendocrine glands responsible for JH production, significantly decreases the likelihood of a mosquito biting, and this can be reversed through the application of JH (Meola and Petralia 1980). Further evidence of the importance of JH in blood feeding is the fact that emerging females entrained into diapause, a dormant state that allows them to survive the winter, have low JH levels (compared to non‐diapausing conspecifics) and forgo blood feeding in favor of increased sugar feeding (Readio et al. 1999; Meola and Petralia 1980). In summation, JH is an important driver of mosquito blood feeding.

High JH levels also prevent immature adult mosquitoes from pupating and ultimately emerge as, if females, blood‐seeking adults (Parthasarathy and Palli 2021). Consequently, juvenile hormone analogs (JHAs), are a standard larvicide. Compounds such as methoprene (Propan‐2‐yl (2E,4E)‐11‐methoxy‐3,7,11‐trimethyldodeca‐2,4‐dienoate), and more recently, pyriproxyfen (4‐Phenoxyphenyl (R/S)‐2‐(2‐pyridyloxy)propyl ether 2‐[1‐(4‐Phenoxyphenoxy)propan‐2‐yloxy]pyridine) are added to water to disrupt regular larval development and pupation. While it is common to apply JHAs as slow release brickettes directly in bodies of water, ultra‐low volume applications of JHAs (sometimes formulated in combination with *Bacillus turingiensis israelensis*) are also used (Maoz et al. 2017). This potentially creates situations in which adult mosquitoes are exposed to JHAs through either contact with treated bodies of water or broadly treated environments. While most research has focused on the effects of JHAs on larval mosquito development, to the best of our knowledge no studies have examined the effects of JHAs on adult blood feeding behavior.

Our objective was to examine the effects of exposure to JHAs on female mosquito blood feeding. We chose a stage expected to be unlikely to blood feed: gravid female *Culex quinquefasciatus*. We tested the effects of direct application of a JHA as well as a simulated environmental exposure on the propensity for a gravid female to take a bloodmeal. In addition, we examined the effects of JHA exposure on egg production and hatchability.

## 2 Materials and Methods

### 2.1 Insects

We purchased 5–7 day old adult *Cx. quinquefasciatus* mosquitoes from Benzon Research Inc. (Carlisle, PA). We selected *Cx. quinquefasciatus* for their readiness to feed on our artificial blood feeding system (Rutledge et al. 1964), ease of observing egg development in females externally, and their importance as a vector species (Negi and Verma 2018). Upon arrival both male and female mosquitoes were transferred to mesh cages and provided 10% sucrose solution ad libitum. These cages were kept inside incubators maintained at 25°C, a relative humidity of approximately 70%, and a photoperiod of 16 L:8D. All experiments were performed during the 8‐h dark period in which *Cx. quinquefasciatus* females actively seek hosts (Bhattacharya et al. 2016).

### 2.2 Juvenile Hormone Application Experiments

**Figure 1:**
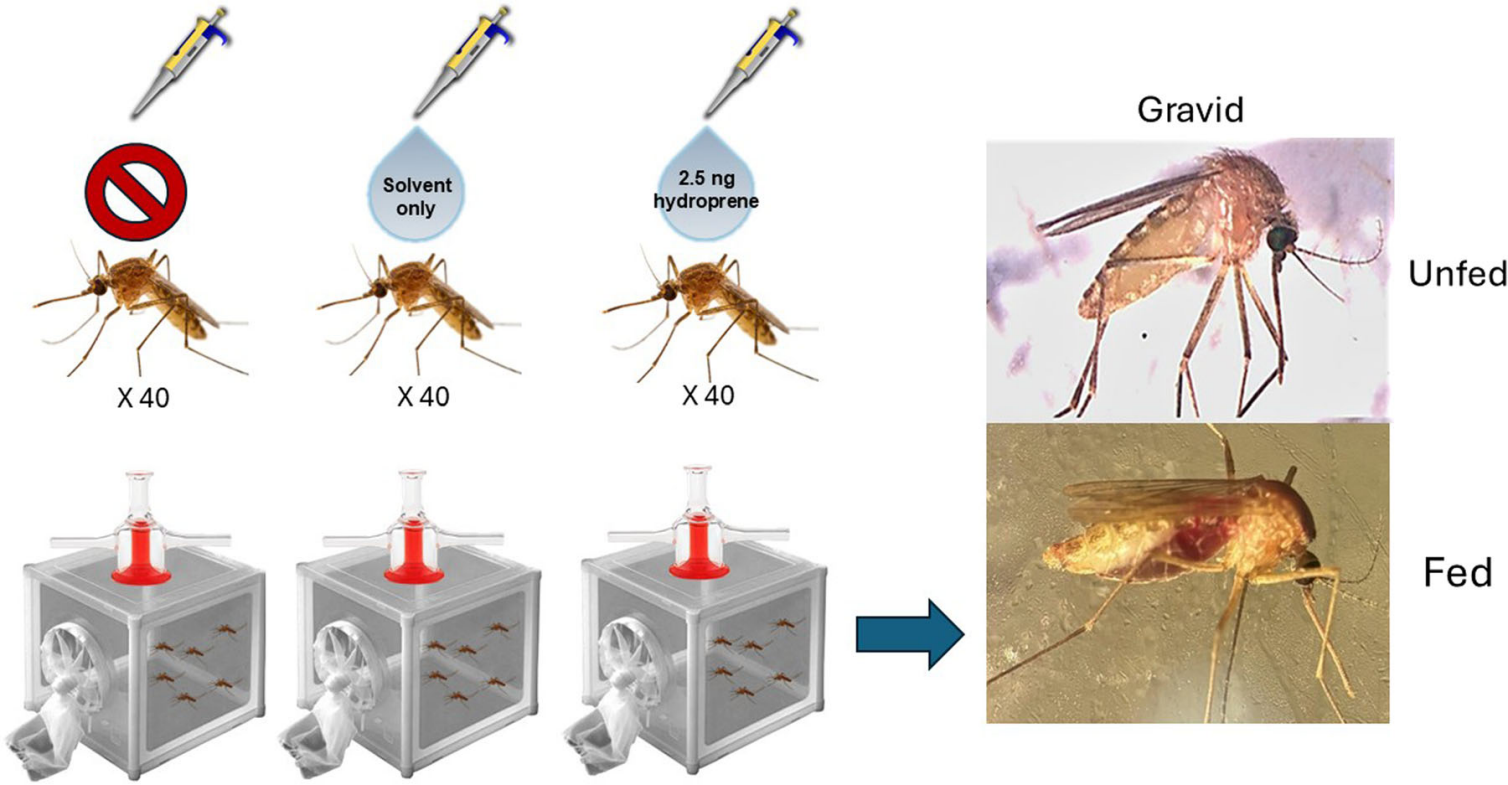
Experimental design of direct s‐hydroprene application to gravid *Culex quinquefasciatus*. We used approximately 40 gravid female mosquitoes per treatment replicate (s‐hydroprene, solvent control, no‐treatment) and provided them blood 24 h after exposure to the treatment. We then collected and counted the number of bloodfed mosquitoes in each group.

We obtained 99% HPLC grade S‐Hydroprene (Sigma‐Aldrich, St Louis, MO), which we added to a 50/50 mixture of acetone and ethanol to a final concentration of 5 ng/µL. This concentration was chosen as it has been shown to cause behavioral shifts in other insects, and was neither the highest nor lowest concentration found in other juvenile hormone related experiments performed in mosquitoes (Spielman 1974). To blood feed, 5–7 day old female *Cx. quinquefasciatus* were given access to an artificial membrane blood feeding system composed of chicken blood in a jacketed reservoir maintained at 35°C by recirculating water. Females were allowed to feed for 1 h or until approximately 80% of them were observed to be engorged. Following blood feeding, females were returned to the incubator and given 48 h to digest the blood and develop eggs. After eggs were visible, and blood could no longer be seen in the abdomens of the mosquitoes, they were then aspirated into groups of approximately 40 individuals and anesthetized by placing them in a freezer for approximately 5 min. After removal from the freezer, we applied 0.5 µL of s‐hydroprene solution to the abdomen of 30 female mosquitoes kept over ice to maintain anesthesia. Following application, we placed the mosquitoes in separate cages based on their treatment group. As controls we treated an additional group of individuals with 0.5 uL of the acetone and ethanol mixture without s‐hydroprene, and another 40 females were left untreated. We placed all individuals in separate cages corresponding to their treatment group and returned them to the incubators for 24 h. Once 24 h had passed, we gave females access to chicken blood through the artificial membrane. After 20 min the mosquitoes were euthanized in a freezer then examined under a microscope to determine the number of gravid females that had taken a bloodmeal. Females were considered gravid if their abdomens were visibly distended with eggs, and eggs could be observed as reaching the 3rd sternite of the abdomen (Figure 1). This threshold was used to exclude any individuals that had initially only taken a partial blood meal. In total, 3 replicates of an average of 42 ± 3.93 (SEM) female mosquitoes were used for each treatment group.

In the first experiment, we randomized the order of the treatments. To assess if JH contamination might be affecting the results we repeated the experiment with all replicates in each treatment group performed separately in succession with the addition of untreated, non‐gravid mosquitoes to compare our experimental groups to normally avid mosquitoes.

### 2.3 Dose Response Experiment

To examine the effects of different concentrations of the s‐hydroprene on gravid female biting, we performed a dose response experiment. Gravid females were treated as described above with 0.25, 2.5, 25, and 250 ng of s‐hydroprene in 0.5 uL of solvent. Three replicates of each concentration were performed using an average of 30 mosquitoes each. Additionally, we counted the number of mosquitoes that died following treatment to quantify relative mortality.

### 2.4 Artificial Flower Exposure

To determine if environmental exposure to JHAs could affect blood feeding behavior, mosquitoes were exposed to grade 2 pore size 8 µm cellulose filter paper (Whatman 10002‐917, Fisher Scientific, Waltham, Massachusetts) treated with s‐hydroprene. Specifically, we cut small strips of treated filter paper measuring approximately 2.5 cm X 0.8 cm from filter paper that was previously saturated in a 5 ng/µL s‐hydroprene solution. The paper strips were allowed to dry at room temperature and then six were attached to a cotton wick that was placed in a 10 mL Erlenmeyer flask containing 10% sucrose solution. The end result resembles a flower. A set of JHA‐untreated “flowers” were also constructed to serve as a control. For the experiment, a single treated or untreated flower was placed in a cage of approximately 150 gravid female mosquitoes. Each cage was placed in an incubator and mosquitoes were allowed interact with the flower for 72 h while sugar feeding. After 72 h, mosquitoes were provided blood as described for the first experiment, and the number of gravid blood‐fed mosquitoes were counted. We repeated the process for four control and four treatment groups.

### 2.5 Effects of a Second Bloodmeal on Egg Number and Hatch Rate

To determine how taking a second bloodmeal while gravid, after JHA exposure, affects egg production, gravid mosquitoes were exposed to the JHA treated filter “flowers” and provided blood as outlined in the previous section. After microscope examination, 30 gravid mosquitoes which had taken a second blood meal, 30 mosquitoes which had not taken a second blood meal, and 30 non‐gravid JHA‐untreated mosquitoes were placed in separate cages inside an incubator kept at 25°C, 70% RH, and 16 L:8D photoperiod. After 48 h, females were provided a container with water for oviposition and 24 h to lay eggs. We collected all egg rafts, counted the number of eggs per raft under a microscope (Olympus Model SZX16), and placed individually in 29.6 ml (1 fluid oz.) plastic containers (Fill‐Rite Corp., Newark, NJ) approximately half filled with water back in the incubator for 24 h to hatch. We counted the number of hatched eggs per raft under the microscope and calculated the proportion of hatched eggs (aka hatch rates) by dividing the number of hatched eggs by the original number of eggs.

### 2.6 Statistical Analysis

All statistical analyses were performed in GraphPad Prism 10.1.2 (GraphPad Software, Boston, Massachusetts, USA). The effects of direct JHA treatment and dose responses were analyzed using a Chi‐square test comparing treatment and both untreated and solvent controls, followed by pairwise comparisons using Fisher's exact test. Mosquito mortality during the dose response experiment was analyzed using ANOVA followed by a Tukey's test for pairwise comparisons. The effect of exposure to JHA treated filter paper flowers was assessed by comparing blood fed gravid females exposed to JHA treated and untreated flowers using Fisher's exact test. The percentages of gravid females taking a bloodmeal between both exposure methods (direct application vs. flower) were compared using a non‐parametric Mann‐Whitney test. Egg numbers produced by nontreated and JHA exposed gravid females that had or had not taken a second flood meal were tested for normality using a Kolmogorov‐Smirnov test and then compared using an ANOVA followed by an uncorrected Fisher's least significant difference test. Respective hatch rates were analyzed using a chi‐square test followed by Fisher's exact test for pairwise comparisons.

## 3 Results

### 3.1 Juvenile Hormone Application Experiments

**Figure 2:**
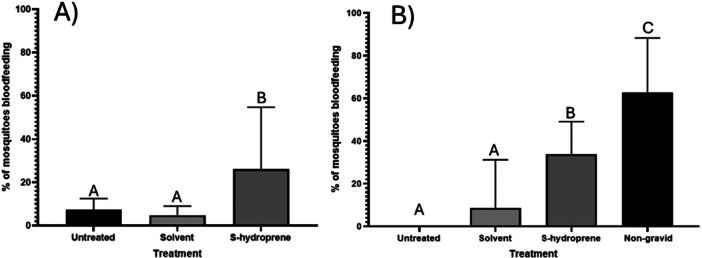
Effects of direct application of s‐hydroprene on number of female *Culex quinquefasciatus* blood feeding while gravid. Data were analyzed using a Chi square test followed by Fisher's exact test for pairwise comparisons. Each bar represents the average number of mosquitoes taking a second bloodmeal across 3 replicates of approximately 40 mosquitoes each. Error bars represent 95% confidence interval. A) Experimental results when replicates within each treatment were randomized. B) Results when each group of replicates was kept separately.

Gravid female mosquitoes exposed to 2.5 ng s‐hydroprene were found to be significantly more likely to take a blood meal when compared to untreated females or females treated with solvent alone (*p* < 0.0001) (Figure 2A). Of note, in pairwise comparisons the treatment was found to be significantly different from both controls (JHA vs solvent *p* < 0.0005, JHA vs untreated *p* < 0.0052) although controls were not found to be significantly different from one another (*p* > 0.9999). In both untreated and solvent controls, a small number of gravid females took a second blood meal.

The repeat experiment with all replicates for each treatment done in tandem (i.e., not randomized) also resulted in a significant difference between treatment groups (*p* < 0.0001) (Figure 2B), but zero female mosquitoes in the untreated control group took a second bloodmeal. Treatment with JHA again caused a significant increase in the proportion of females taking a second blood meal compared to the controls (JHA vs solvent *p* < 0.0001, JHA vs untreated *p* < 0.0001). In addition, the acetone control was significantly different from the untreated controls (*p* = 0.0005). Non‐gravid untreated mosquitoes fed at a statistically higher rate than all gravid groups (non‐gravid vs solvent *p* < 0.0001, non‐gravid vs untreated *p* < 0.0001, non‐gravid vs JHA *p* < 0.0001).

### 3.2 Dose Response Experiment

**Figure 3:**
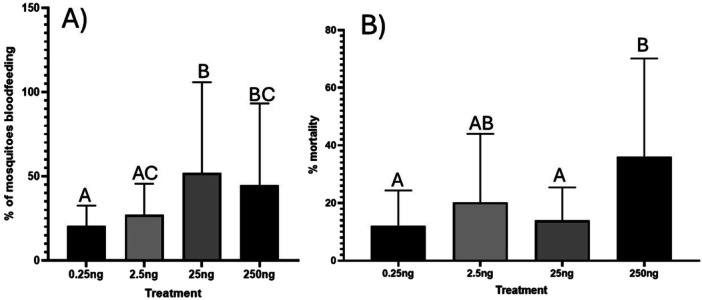
Results of dose response experiment. A) Blood feeding response of gravid females to differing amounts of s‐hydroprene. Data were analyzed using a Chi square test followed by Fisher's exact test for pairwise comparisons. Bars represent 95% confidence interval. B) Mortality of mosquitoes exposed to differing amounts of s‐hydroprene. Data were analyzed using ANOVA followed by Tukey's test for pairwise comparisons. Bars represent 95% confidence interval.

Increasing amounts of s‐hydroprene were found to have statistically significant effects on the number of gravid females taking a bloodmeal (*p* < 0.0001) (Figure 3A). Application of 0.25 ng was not statistically different from 2.5 ng (*p* = 0.2574), but both 25 and 250 ng caused a statistically significant increase in rates of blood feeding (*p* < 0.0001, *p* = 0.0040). 25 ng was found to have increased blood feeding rates in comparison to 2.5 ng (*p* = 0.0073), but 2.5 ng was not found to be statistically different from 250 ng (*p* = 0.0944). Applications of 25 ng were also not found to be statistically different from 250 ng (*p* = 0.4788). Additionally, differences in concentration applied did cause statistically different levels of mortality within treatment groups (*p* = 0.0252) (Figure 3B). Pairwise comparisons revealed a significant difference in mortality between the 0.25 and 250 ng groups (0.0266) and between the 25 and 250 ng groups (0.0454).

### 3.3 Artificial Flower Exposure

**Figure 4:**
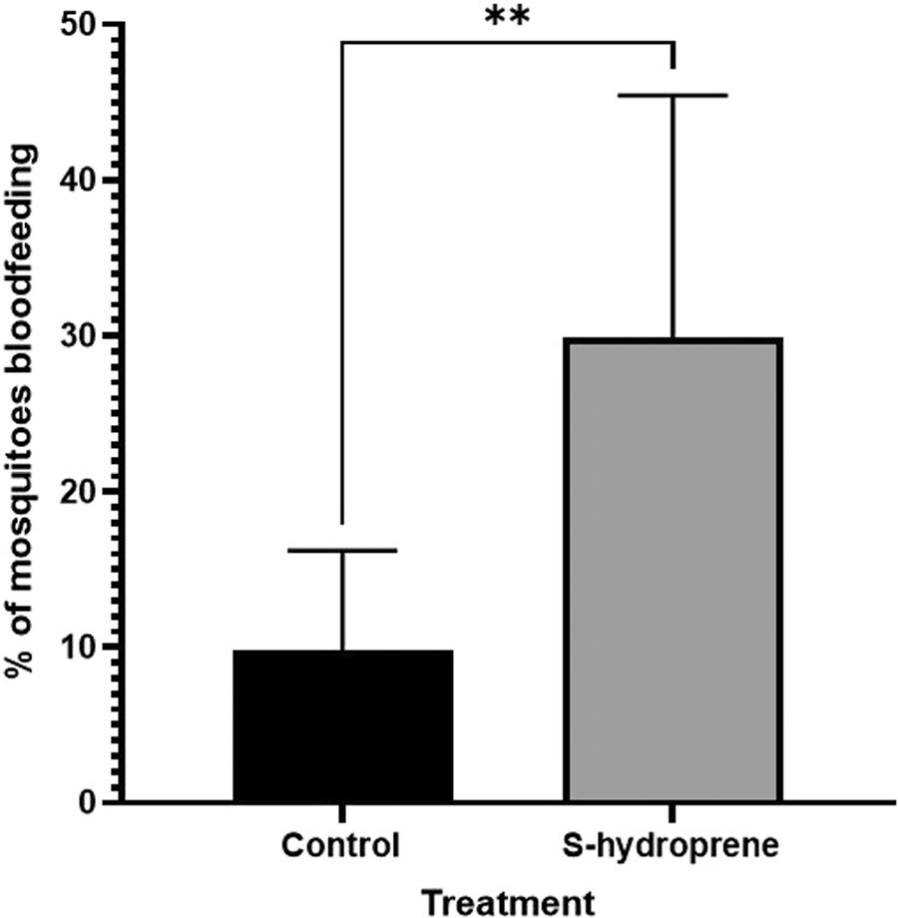
Effects of exposure to s‐hydroprene treated filter paper flowers on number of female *Culex quinquefasciatus* blood feeding while gravid. Data were analyzed using Fisher's exact test. Bars represent 95% confidence interval.

After exposure to JHA treated filter paper flowers, gravid mosquitoes were significantly more likely to take a second bloodmeal compared to mosquitoes exposed to untreated flowers (*p* < 0.0001) (Figure 4). There was no significant difference in the percentage of gravid females taking a bloodmeal after exposure to treated flowers compared to direct applications of JHA (*p* > 0.9999).

### 3.4 Effects of Second Bloodmeal on Egg Number and Hatch Rate

**Figure 5:**
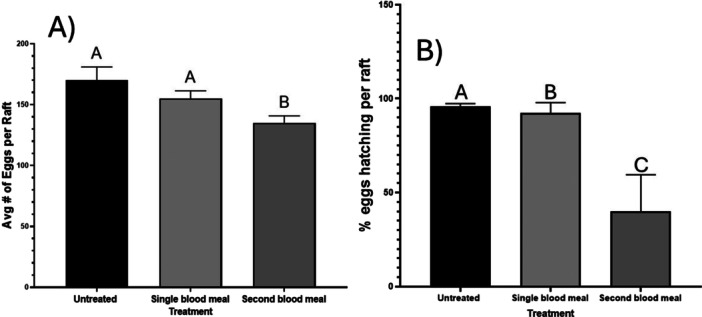
Egg numbers and hatch numbers per raft laid by female *Culex quinquefasciatus* that took an additional bloodmeal while gravid in comparison to gravid female *Cx. quinquefasciatus* that did not. Gravid females in both treatments had been exposed to JHAs. A) Average egg numbers per raft. Data were analyzed using ANOVA followed by an uncorrected Fisher's least significant difference test. B) Average number of hatched eggs per raft. Data were analyzed using a chi‐square test followed by Fisher's exact test for pairwise comparisons. In both A and B bars represent 95% CI.

We found a significant difference in the number of eggs laid by gravid mosquitoes, JHA treated gravid mosquitoes, and JHA treated mosquitoes which took a second bloodmeal (*p* = 0.0006). Post‐hoc comparisons found that treated mosquitoes taking a second bloodmeal laid a significantly lower number of eggs compared to untreated mosquitoes (*p* = 0.0001), and treated mosquitoes that did not take a second bloodmeal (*p* = 0.0232). Of note, we did not find a difference between JHA treated females and untreated females (*p* = 0.062). (Figure 5A). The number of hatched eggs was also significantly different among groups (*p* < 0.0001) (Figure 5B). Eggs from untreated mosquitoes hatched at a higher rate than both treated mosquitoes (*p* < 0.0001) and treated mosquitoes with a second bloodmeal (*p* < 0.0001). Taking a second blood meal also lowered the number of hatched eggs in comparison to treated mosquitoes which had not taken a second bloodmeal (*p* < 0.0001). Approximately 96% of untreated mosquito eggs hatched, while 90% of eggs hatched from gravid females that did not take a second bloodmeal, and only 61% of eggs hatched from rafts laid by females taking a second bloodmeal.

## 4 Discussion

We found that exposure to juvenile hormone analogs significantly increases the blood avidity of gravid female mosquitoes. Several studies have examined the role of JH in blood feeding and found this hormone is critical to both egg development and host seeking (Readio et al. 1999; Meola and Petralia 1980; Shiao et al. 2008). Because blood feeding must follow early egg development, it is difficult to separate the effects of pre‐vitellogenic egg development from the action of JH in initiating blood feeding. It could be hypothesized that once nutrients from a bloodmeal can be used for egg development, another endogenous signal produced by the eggs could initiate the blood feeding behavior of mosquitoes, as studies examining this behavior typically involve younger mosquitoes which have not taken a bloodmeal (Hancock and Foster 2000; Alto et al. 2003). By using gravid females which are typically inhibited from blood feeding, we have further supported that JH signaling is directly involved in the activation of blood avidity.

We found that treating mosquitoes with increasing concentrations of s‐hydroprene up to 25 ng corresponded with increasing rates of gravid females taking a second bloodmeal. While it is difficult to determine the actual physiological dose of JHA needed to cause abnormal blood feeding behavior (i.e., the amount i.e. absorbed), applications of 25 ng led to an average of 51% of mosquitoes taking a second bloodmeal. We also found high mortality in mosquitoes treated with 250 ng of JHA, a result that we are currently at a loss to understand but hypothesize may be due to crosstalk between JH and nutritional signaling pathways causing disruption of normal metabolic function (Hansen et al. 2014; Sharma et al. 2019).

There was no statistical difference between the percentages of gravid females that blood fed after direct applications of JHA or exposure to JHA treated flowers. This was surprising as the prolonged exposure period associated with the treated flower experiment was expected to result in a greater exposure to JHA. Additionally, JHA from the “flowers” likely leached into the sugar solution in the Erlenmeyer and resulted in the females ingesting JHA while drinking. However, we did not find an effect of route or length of exposure to JHAs to blood feeding behavior.

After finding that a few gravid females in the untreated group and acetone‐ethanol control in the first experiment took a second blood meal we suspected contamination with JH across treatments, as hormones are known to act at very small concentrations. Therefore, in a second experiment, we performed all replicates for each treatment separately starting with the controls, and found that zero females in the untreated controls fed a second time when gravid. This corroborates our original hypothesis that environmental contamination of JHAs may have caused some untreated individuals in our control groups to take a second bloodmeal. In contrast, in the second experiment we still found that some individuals in the acetone‐ethanol treatment group took a second bloodmeal. We hypothesize this may be due to the solvent itself stripping some components of the cuticle away, resulting in increased desiccation. Dehydration has been shown to increase the likelihood of a mosquito taking additional bloodmeals (Hagan et al. 2018; Holmes et al. 2022). Even considering the possible cross‐treatment contamination, in both experiments the treatment with s‐hydroprene produced a significantly higher proportion of female mosquitoes taking a second bloodmeal while gravid. In addition, in the flower exposure treatments neither group of mosquitoes were exposed to solvents, and there was still a significant increase in the likelihood that gravid females would take a second bloodmeal in the s‐hydroprene treatment, negating the possibly that dehydration per se, led to gravid females’ blood feeding.

It is difficult to argue that the levels of JHA we used are representative of the levels mosquitoes might be exposed in the field without detailed calculations of field application rates, degradation rates, etc. The literature on the subject is unclear and likely varies between mosquito control programs. As mentioned previously, while these compounds are typically applied as briquettes or other solid formulations, increasingly they are being applied as sprays (Suman et al. 2014; Doud et al. 2014). In addition, pyriproxyfen is commonly used in auto‐dissemination approaches (Kancharlapalli et al. 2022; Kancharlapalli et al. 2021). Our results reveal potential drawbacks of using these compounds as larvicides and warrants further studies on the degree to which adult mosquitoes may be exposed to JHAs in nature.

Interestingly, we found that taking a second bloodmeal while gravid significantly decreased the number of eggs laid and their hatch rate. Of note, the numbers of eggs and hatch rates of untreated and JHA exposed mosquitoes that did not take a blood meal are within the averages reported in the literature for *Cx. quinquefasciatus* (Oda et al. 1980; Mccann et al. 2009). Other studies have shown the detrimental effects of JHAs on egg development in both adults exposed before blood feeding and eggs exposed to JHA directly (Klowden and Chambers 1989; Brabant and Dobson 2013; Suman et al. 2013). In *Aedes aegypti*, treatment with JHAs around 36 h after taking a bloodmeal was found to have the most significant reduction (up to 90%) in reproductive output with the effect diminishing with increasing time post‐bloodmeal(Patterson 1974). Our results agree with this, as mosquitoes were treated at a minimum of 48 h following the initial bloodmeal and we only saw an average reduction of approximately 6% between treated and nontreated gravid mosquitoes. Importantly, we found a much greater decrease in reproductive output from gravid *Cx. quinquefasciatus* exposed to s‐hydroprene if they took a second blood meal. The fact that the declines in eggs and hatch rate were limited to those females that took another blood meal, indicates the blood was not used to develop more eggs.

The acquisition of blood and the following development of eggs are associated with distinct states in behavior and hormonal signaling (Zhu and Noriega 2016; Hansen et al. 2014). Blood acquired when females would have been inhibited from feeding may interfere with processes involved in egg retention and maintenance. Eggs retained by female *Culex* mosquitoes are a considerable mass of metabolically active tissues in relation to the total body weight of the mosquito. Taking a bloodmeal is also well understood to initiate genome wide transcriptional changes involved in digestion, egg development, and protection from harmful compounds such as heme (Gracasouza et al. 2006; Bonizzoni et al. 2011). To metabolize a new bloodmeal, gravid mosquitoes may need to reallocate resources that would normally be used to maintain already developed eggs resulting in lower hatch rates and egg numbers. Further studies investigating the effects of a second bloodmeal on gene expression might determine which genes are responsible for egg maintenance once developed.

Juvenile hormone analogs remain a common larvicide for control of mosquito populations. As its name suggests, the majority of what is known about JH and JHAs is related to the immature stages of insects and JH's role in regulating metamorphosis and development and how exposure to these compounds can be used to human benefit (Jindra et al. 2013; Riddiford 2008; Parthasarathy and Palli 2021). However, it is important to be aware that JH is also produced in adult insects and has been shown to regulate a wide variety of processes and behaviors across insect taxa (Hartfelder 2000; Tibbetts et al. 2020; Dingle and Winchell 1997). While the role of JH in mosquito development and reproduction is relatively well understood, our results provide additional insight into how JH regulates epidemiologically relevant behavior and offers a useful model system for further study.

## Author Contributions

**Grayson A. Tung:** conceptualization, investigation, writing – original draft, methodology, visualization, writing – review and editing, data curation, funding acquisition. **Dina M. Fonseca:** writing – review and editing, supervision, resources, funding acquisition.

## Conflicts of Interest

The authors declare no conflicts of interest.

## Data Availability

The data that support the findings of this study are available from the corresponding author upon reasonable request.
